# The Association between the Cross-Sectional Area of the Dural Sac and Low Back Pain in a Large Population: The Wakayama Spine Study

**DOI:** 10.1371/journal.pone.0160002

**Published:** 2016-08-03

**Authors:** Hiroki Iwahashi, Noriko Yoshimura, Hiroshi Hashizume, Hiroshi Yamada, Hiroyuki Oka, Ko Matsudaira, Kazunori Shinto, Yuyu Ishimoto, Keiji Nagata, Masatoshi Teraguchi, Ryohei Kagotani, Shigeyuki Muraki, Toru Akune, Sakae Tanaka, Hiroshi Kawaguchi, Kozo Nakamura, Akihito Minamide, Yukihiro Nakagawa, Munehito Yoshida

**Affiliations:** 1 Department of Orthopaedic Surgery, Wakayama Medical University, 811–1 Kimiidera, Wakayama City, Wakayama 641–8510, Japan; 2 Department of Joint Disease Research, 22nd Century Medical and Research Center, Faculty of Medicine, The University of Tokyo, 7-3-1 Hongo, Bunkyo-ku, Tokyo 113–8655, Japan; 3 Department of Medical Research and Management for Musculoskeletal Pain, 22nd Century Medical & Research Center, Faculty of Medicine, The University of Tokyo, 7-3-1 Hongo, Bunkyo-ku, Tokyo 113–8655, Japan; 4 Rehabilitation Services Bureau, National Rehabilitation Center for Persons with Disabilities, 1 Namiki 4-chome, Tokorozawa City, Saitama 359–8555, Japan; 5 Department of Orthopaedic Surgery, Faculty of Medicine, The University of Tokyo, 7-3-1 Hongo, Bunkyo-ku, Tokyo 113–8655, Japan; 6 Japan Community Health Care Organization Tokyo Shinjuku Medical Center, 6-1-1 Shinjuku, Shinjuku-ku, Tokyo 160–8402, Japan; The University of Tokyo Hospital, JAPAN

## Abstract

**Objective:**

The purpose of this study was to evaluate the relations between the degree of encroachment, measured as the cross-sectional area of the dural sac, and low back pain in a large population.

**Methods:**

In this cross-sectional study, data from 802 participants (247 men, 555 women; mean age, 63.5 years) were analyzed. The measurement of the cross-sectional area of the dural sac from the level of L1/2 to L4/5 was taken using axial T2-weighted images. The minimum cross-sectional area was defined as the cross-sectional area of the dural sac at the most constricted level in the examined spine. Participants were divided into three groups according to minimum cross-sectional area measurement quartiles (less than the first quartile, between the first and third quartiles, and greater than the third quartile). A multivariate logistic regression analysis was used to estimate the association between the minimum cross-sectional area and the prevalence of low back pain.

**Results:**

The mean minimum cross-sectional area was 117.3 mm^2^ (men: 114.4 mm^2^; women: 118.6 mm^2^). A logistic regression analysis adjusted for age, sex, body mass index, and other confounding factors, including disc degeneration, showed that a narrow minimum cross-sectional area (smaller than the first quartile) was significantly associated with low back pain (odds ratio, 1.78; 95% confidence interval, 1.13–2.80 compared to the wide minimum cross-sectional area group: minimum cross-sectional area greater than the third quartile measured).

**Conclusion:**

This study showed that a narrow dural sac cross-sectional area was significantly associated with the presence of low back pain after adjustment for age, sex, and body mass index. Further investigations that include additional radiographic findings and psychological factors will continue to elucidate the causes of low back pain.

## Introduction

Low back pain (LBP) is a multifactorial symptom, a common cause of morbidity and disability, and was reported to have a prevalence of 28.5% in a recent study [1,2]. There are many causes of chronic LBP, one of which is lumbar spinal stenosis (LSS) [3]. According to the Evidence-Based Clinical Guidelines for Multidisciplinary Spine Care developed by The North American Spine Society [4], degenerative LSS describes a condition in which there is diminished space available for the neural and vascular elements in the lumbar spine secondary to degenerative changes in the spinal canal. When symptomatic, this condition causes a variable clinical syndrome of gluteal and/or lower-extremity pain and/or fatigue, which may occur with or without back pain. In reality, however, 67.5%–95% of patients with LSS experience LBP [5–7].

LBP in patients with LSS is also multifactorial. Patients with LSS often have facet arthrosis and degenerative discs. These pathologies may explain their back pain. Earlier findings from preoperative imaging studies of patients with central spinal stenosis have suggested that the cross-sectional area (CSA) of the dural sac was closely related to preoperative walking ability, health-related quality of life, leg pain, and LBP [8–10]. Recent studies have also reported the possibility of improving LBP following decompression surgery [11,12]. Thus, it is possible that constriction of the dural sac is also the cause of LBP in LSS patients.

However, no research to date has focused on the association between the prevalence of LBP and the cross-sectional area of the dural sac in the general population. Thus, the purpose of this study was to evaluate the relations between the degree of encroachment, measured as the CSA of the dural sac, and LBP in a large population.

## Methods

### Study design

We performed a cross-sectional, population-based study.

### Participants

**Fig 1:**
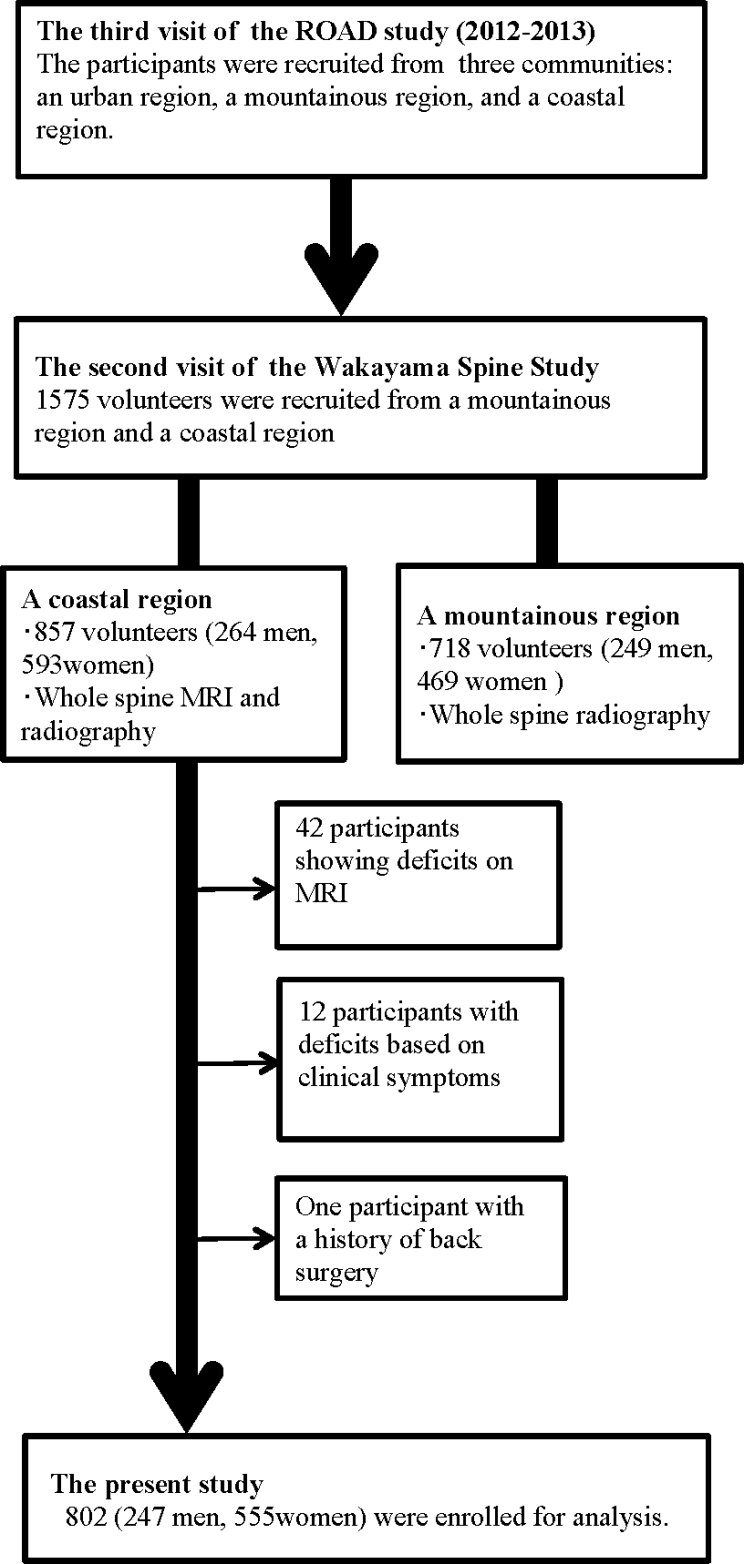
Flow diagram depicting participants recruited to the present study from the third visit of the ROAD study.

The present study design was approved by the Wakayama Medical University Ethics Committee. All participants provided their written informed consent. The Wakayama Spine Study is a population-based study of degenerative spinal disease [13–17] conducted with a sub-cohort of the large-scale, population-based cohort study Research on Osteoarthritis/Osteoporosis against Disability (ROAD) [18,19]. ROAD is a nationwide, prospective study of bone and joint diseases consisting of population-based cohorts established in three communities in Japan. The participants were recruited from listings of resident registrations in three communities that have different characteristics: an urban region in I town, Tokyo; a mountainous region in H town, Wakayama; and a coastal region in T town Wakayama. The inclusion criteria, apart from residing in those communities, included the ability to walk to the survey site, to report data, and to understand and sign an informed consent form. No other exclusion criteria were used. A third visit of the ROAD study began in 2012 and was completed in 2013. From the volunteers participating in the third visit of the ROAD study, 1575 individuals (513 men, 1062 women), which included 718 individuals in the mountainous area and 857 individuals in the coastal area, were recruited to the second visit of the Wakayama Spine Study. Magnetic resonance imaging (MRI) was conducted only to the individuals in the coastal area because of the funding limitation. Thus, we evaluated data from 857 individuals in the coastal area for the present study. Among them, 42 participants with incomplete MRI records and one participant who had previously undergone posterior lumbar fusion were excluded from the analysis (Fig 1).

Experienced board-certified orthopedic surgeons also asked all participants the following question regarding LBP and buttock and leg pain: ‘‘Have you experienced LBP (or buttock and leg pain) on most days during the past month, in addition to now?” Those who answered ‘‘yes” were defined as having LBP (buttock and leg pain), based on previous studies [20–24]. Twelve participants who lacked information regarding LBP or buttock and leg pain were excluded. Thus, 802 participants (247 men and 555 women) ranging in age from 19 to 93 years (mean, 63.0 years for men and 63.8 years for women) were included for analysis (Fig 1). All study participants provided informed consent, and the study design was approved by the appropriate ethics review boards.

### Magnetic resonance imaging

A mobile MRI unit (Achieva 1.5 T; Philips Medical Systems, Best, the Netherlands) was used, and whole-spine MRI was performed for all participants on the same day as the examination. The participants were supine during the MRI, and those with rounded backs used triangular pillows under their heads and knees. The imaging protocol included sagittal T2-weighted fast spin echo imaging (repetition time, 3,000 ms/echo; echo time, 120 ms; and field of view, 270 × 270 mm) and axial T2-weighted fast spin echo imaging (repetition time, 2,100 ms/echo; echo time, 100 ms; and field of view, 180 × 180 mm). Sagittal images were taken for the entire spine, but axial images were obtained for each lumbar intervertebral level (L1/2–L5/S1) parallel to the vertebral endplates.

### The CSA of the dural sac

**Fig 2:**
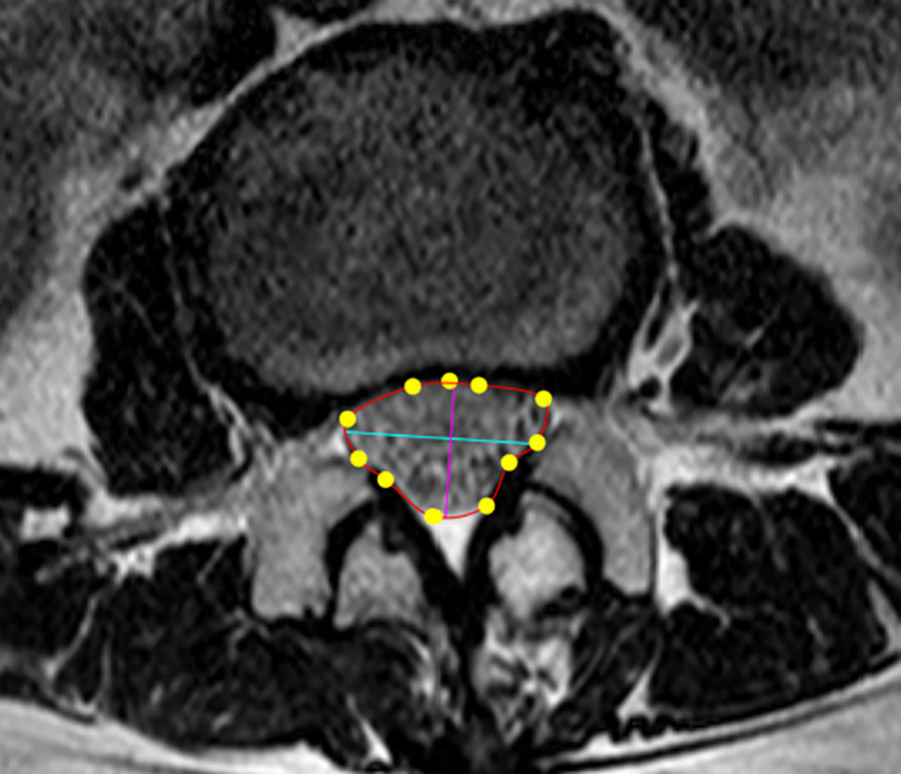
Illustration of the dural sac cross-sectional area measurement technique.

CSA measurement was performed with axial T2-weighted images using a radiological workstation specially designed for such purposes. The CSA of the lumbar dural sac, defined as the area occupied by the dural sac at the disc level, was measured from the level of L1/2 to L4/5 (Fig 2). The measurement was performed by an orthopedic surgeon who was blinded to the background of the participants. The CSA of the dural sac at the most constricted level in the examined spine was called the minimum CSA (mCSA) [8]. The participants were divided into three groups according to quartiles (the narrow group: mCSA less than the first quartile [Q1] measurement; the middle group: mCSA between the Q1 and the third quartile [Q3] measurements; and the wide group: mCSA greater than the measurement for Q3).

### Disc degeneration

Disc degeneration (DD) grading was performed by a board-certified orthopedic surgeon who was blinded to the background of the participants, in accordance with previous studies. The degree of DD on MRI was classified into five grades based on the Pfirrmann classification system [25], with grades 4 and 5 indicating DD.

The signal intensity for grade 4 was intermediate to hypointense for the cerebrospinal fluid (dark gray), while the structure was inhomogeneous. Meanwhile, for grade 5, the signal intensity was hypointense for the cerebrospinal fluid (black), and the structure was likewise inhomogeneous. In addition, the disc space was collapsed

### Statistical analysis

Radiographic changes were compared between sexes using the chi-squared test. The Jonckheere-Terpstra test was used to identify trends with regard to age or spinal levels for the CSA of dural sac. To test the association between the presence of LBP and mCSA, we used the Cochran-Armitage test and multiple logistic regression analysis. In the regression analysis, we used the presence or absence of LBP as the objective variable and mCSA (the wide group vs. the narrow group; the wide group vs. the middle group) and the presence or absence of DD and buttock and leg pain as explanatory variables, in addition to basic characteristics, such as age, sex, and body mass index (BMI). Receiver operator characteristic (ROC) curves and the corresponding areas under the curve (AUCs) were used to evaluate how the prediction model performed on the test data. The AUC was also calculated independently for the factors in the final model to demonstrate the additional value gained from the addition of each factor to the model. ROC curves plot the true-positive rate (sensitivity) vs the false-positive rate (1-specificity). All statistical analyses except Jonckheere-Terpstra test were performed using JMP, version 8 (SAS Institute Japan, Tokyo, Japan). The Jonckheere-Terpstra test was performed using SPSS statistics 23 (IBM Japan, Tokyo, Japan).

## Results

Table 1 shows the characteristics of the 802 participants in the present study, including age, demographic measurements, and symptoms. The prevalence of LBP and buttock and leg pain was 38.6% and 23.3%, respectively. The mean mCSA was 117.3 mm^2^ (men: 114.4 mm^2^, women: 118.6 mm^2^). Q1 and Q3 values for mCSA for the group overall were 85.8 mm^2^ and 147.2 mm^2^, respectively. The proportion of each group divided into quartiles based on the mCSA values among the 802 participants was as follows: for men, 30.0% for the narrow group (mCSA ≤ Q1), 48.2% for the middle group (Q1 < mCSA ≤ Q3), and 21.8% for the wide group (Q3 < mCSA), and for women, 22.9% for the narrow group, 50.8% for the middle group, and 26.3% for the wide group. The proportion of men in the narrow group was significantly higher than the proportion of women in the narrow group (p = 0.032). The prevalence of disc degeneration in the 802 participants was 91.4% (men: 91.1%, women: 91.5%). There were no significant differences for the prevalence of DD between men and women.

**Table 1:** Characteristics of participants.

	Overall	Men	Women
**No. of participants**	**802**	**247**	**555**
**Demographic characteristics**			
Age (years)	63.5±13.1	63.0±13.9	63.8±12.7
Height (cm)	157.4±8.9	166.8±6.8	153.4±6.4
Weight (kg)	57.3±11.4	66.7±10.8	53.0±8.9
Body mass index (kg/m^2^)	23.0±3.6	24.0±3.5	22.6±3.6
**Symptom**			
Low back pain	309(38.6%)	94(38.2%)	215(38.7%)
Buttock and leg pain	186(23.3%)	47(19.1%)	139(25.0%)

Data are presented as means ± standard deviation or as n (%).

Table 2 summarizes the distribution of the CSA of the dura of the 802 participants in the present study. A Jonckheere-Terpstra test for ordered alternatives showed that there was a statistically significant trend of smaller median CSAs with higher age strata at all intervertebral levels from L1/2 to L4/5 in both genders (p < 0.0005). The CSAs had a tendency to decrease with lower intervertebral levels in both genders (p < 0.0005).

**Table 2:** Distribution of cross sectional area of dura (mm^2^).

**Men**	**Total**	**<50**	**50–59**	**60–69**	**70–79**	**≧80**	**Standardized Test Statistic**	**p-value**
**L1/2**	172[149–192]	192[174–212]	177[154–193]	170[147–189]	154[132–181]	157[147–186]	-4.619	<0.0005
**L2/3**	146[120–172]	176[151–187]	157[136–174]	143[117–163]	126[107–158]	142[109–165]	-5.246	<0.0005
**L3/4**	132[102–165]	164 [146–181]	144[122–178]	122[102–149]	114[86–140]	119[88–137]	-5.652	<0.0005
**L4/5**	129[91–168]	166 [128–198]	140[111–186]	120 [88–157]	113[90–146]	99[73–154]	-5.538	<0.0005
**Women**	**Total**	**<50**	**50–59**	**60–69**	**70–79**	**≧80**	**Standardized Test Statistic**	**p-value**
**L1/2**	177[157–202]	204[176–220]	186[165–205]	176[156–199]	165[147–189]	168[145–191]	-7.506	<0.0005
**L2/3**	158[133–184]	188[168–210]	170[147–190]	153[130–178]	145[123–169]	148[112–161]	-8.915	<0.0005
**L3/4**	139[109–174]	179[149–197]	154[126–177]	134[107–163]	126[97–163]	113[73–153]	-8.775	<0.0005
**L4/5**	127[96–166]	149[111–189]	140[109–166]	123[96–157]	112[83–160]	107[77–141]	-6.003	<0.0005

Values are the median [first quartile- third quartile].

The CSAs had a tendency to decrease with age and lower intervertebral levels in both genders (Jonckheere-Terpestra test; p< 0.0005).

On analyzing the relationship between the prevalence of LBP and mCSA, we found that the prevalence of LBP increased as mCSA decreased. The prevalence of LBP was 50.3% for the narrow group, 36.6% for the middle group, and 30.8% for the wide group. The participants who had narrower mCSA values were more likely to have LBP (p < 0.0001).

Logistic regression analyses were performed with LBP as the objective variable, mCSA as the explanatory variable, and patient characteristics, including age, sex, and BMI, as potential risk factors (model 1). Belonging to the middle group (Q1 < mCSA ≤ Q3) was not significantly associated with LBP (odds ratio, [OR] 1.26; 95% confidence interval [CI], 0.87–1.82). On the other hand, belonging to the narrow group (mCSA ≤ Q1) was significantly associated with LBP (OR, 1.97; 95% CI, 1.27–3.04).

We then added the presence of DD as a dependent variable (model 2). Belonging to the middle group (Q1 < mCSA ≤ Q3) was not significantly associated with LBP (OR, 1.19; 95% CI, 0.82–1.74). In contrast, belonging to the narrow group (mCSA ≤ Q1) was significantly associated with LBP (OR, 1.94; 95% CI, 1.25–3.02).

Finally, the presence of buttock and leg pain was added as a dependent variable (model 3). Belonging to the middle group (Q1 < mCSA ≤ Q3) was not significantly associated with LBP (OR, 1.18; 95% CI, 0.80–1.73); however, belonging to the narrow group (mCSA ≤ Q1) was significantly associated with LBP (OR, 1.78; 95% CI, 1.13–2.80). The results of the logistic regression analysis for all models are summarized in Table 3.

**Table 3:** Association between low back pain and the minimum cross-sectional area in each logistic regression model.

	Explanatory variables	Category	OR	95%CI	AUC
**model 1**	mCSA	mCSA<Q1 vs. mCSA>Q3	2.02	1.30–3.12	0.59
		Q1≦mCSA<Q3 vs. mCSA>Q3	1.26	0.87–1.82	
**model 2**	mCSA	mCSA<Q1 vs. mCSA>Q3	1.94	1.25–3.02	0.6
		Q1≦mCSA<Q3 vs. mCSA>Q3	1.2	0.82–1.74	
	DD	1:presence 0:absence	2.41	1.23–4.73	
**model 3**	mCSA	mCSA<Q1 vs. mCSA>Q3	1.78	1.13–2.81	0.66
		Q1≦mCSA<Q3 vs. mCSA>Q3	1.18	0.80–1.73	
	DD	1:presence 0:absence	2.38	1.20–4.72	
	buttock and leg pain	1:presence 0:absence	3.31	2.33–4.69	

CI, confidence interval; DD, disc degeneration; mCSA, minimum cross-sectional area; OR, odds ratio; Q1, the first quartile; Q3, the third quartile; AUC, areas under the curve

Note: Multivariate logistic regression analysis of mCSA was associated with low back pain after adjustment for age, body mass index, and sex in each model. The minimum cross-sectional area of the dural sac is the cross-sectional area of the dural sac at the most constricted level in the examined spine from the level of L1/2 to L4/5. Q1, 85.8 mm2; median, 114.2 mm2; Q3, 147.2 mm

**Fig 3:**
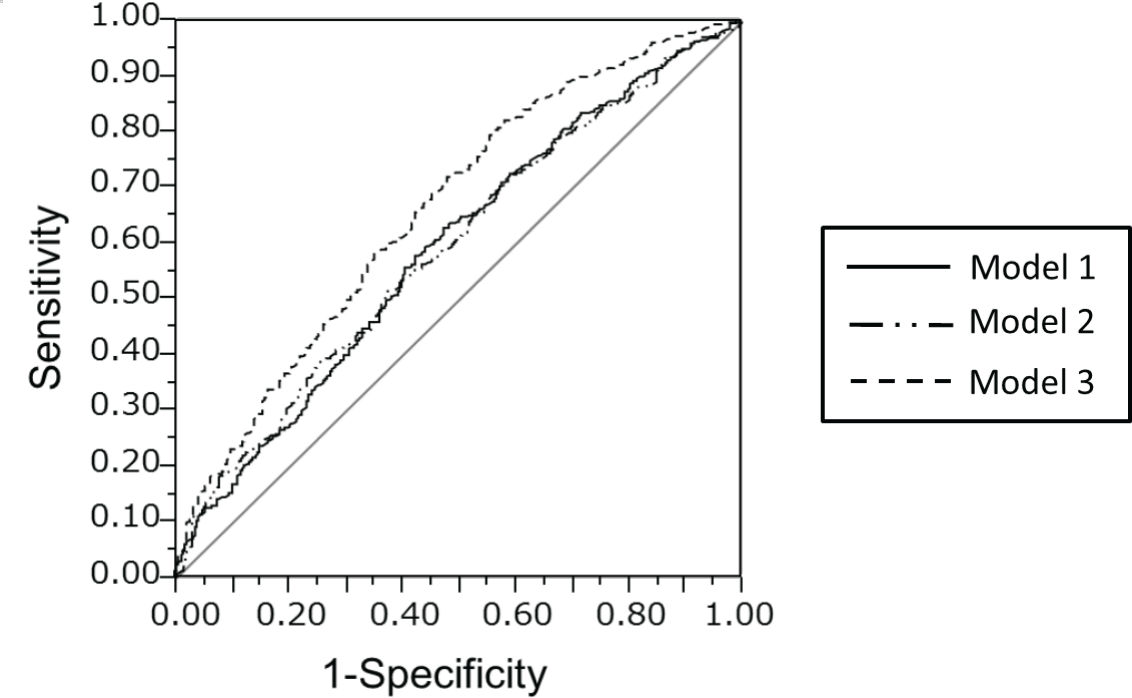
Receiver operating characteristic (ROC) curves for the multiple logistic regression models for low back pain. The area under the curve (AUC) for the ROC curves for models 1, 2, and 3 were 0.59, 0.60, and 0.66, respectively. The AUC for model 3 was significantly greater than the AUCs for models 1 and 2.

Fig 3 shows the receiver operating characteristic (ROC) curves for the multiple logistic regression models for LBP. The AUC for model 1 was 0.59; for model 2, 0.60; and for model 3, 0.66. The AUC for model 3 was significantly higher than those for the other two models (p = 0.0008).

## Discussion

The purpose of this study was to evaluate the relations between the degree of encroachment, measured as the CSA of the dural sac, and LBP in a large population. In this study, narrowed dural sac CSA was significantly associated with the presence of LBP after adjustment for age, sex, and BMI. To the best of our knowledge, this is the first report of a positive association between LBP and the CSA of the dural sac in a large population of individuals ranging in age from 19 to 93 years old.

There are two possibilities to explain the narrow CSA of the dural sac. The narrow dural sac could be due to degenerative changes or to developmental stenosis of the dural sac. In this study, we considered that a narrow CSA of the dural sac was related to constriction of the dural sac due to degenerated tissue around the dural sac rather than to congenital stenosis, as 65.2% of the participants were older than 60 years, and in fact, almost all patients with a small dural sac CSA had other degenerative findings, such as a bulging disc, thickened facet joint, and/or ligamentum flavum.

Findings from earlier preoperative imaging studies on patients with central spinal stenosis have suggested that the CSA of the dural sac was closely related to preoperative walking ability, health-related quality of life, leg pain, and LBP [8,10,26,27]. Moreover, it has been reported that LBP significantly improves following spinal decompression alone [12]. Jones et al. investigated Visual Analog Scale data for LBP in 119 patients with LSS, and they reported that there was a significant reduction in mean LBP from baseline to 6 weeks and 1 year postoperatively. Spinal decompression surgery has long been considered the gold standard surgical treatment for symptomatic LSS. The aim of decompression surgery is to improve radicular leg pain and walking distance. The authors concluded that there is a possibility for improvement in LBP after decompression surgery. We believe our current findings from an established, population-based cohort support the conclusions of the Jones et al. report. However, future studies are needed in order to identify patients who will show improvement in LBP after decompression surgery.

A potential explanation for LBP in LSS patients is the reduction in nutrient supply to ischemic nerves and hence the development of claudication pain originating from muscles supplied by the dorsal rami at the stenotic level [28]. Moreover, Konnai et al. reported that the lower lumbar dura mater is innervated by sensory nerves derived from upper lumbar dorsal root ganglia via the lumbar sympathetic trunk in rats. They concluded that these sensory nerves may mediate LBP and possibly interact with sympathetic nerves [29]. In LSS patients, the dural sac is encroached by degenerative tissue, such as a thickened ligamentum flavum, bulging disc, or osteophyte. Thus, sensory nerves innervating the dural sac can be pinched by degenerative tissues, which might cause LBP in LSS patients.

In the present study, disc degeneration was added as an objective variable to the multivariable regression model. As mentioned above, degenerative discs might be a potential source of LBP in LSS patients. Teraguchi et al. showed that there was a significant positive association between the presence of DD in the lumbar region and LBP [14]. According to that study, the presence of DD in the lumbar region was significantly associated with LBP. Thus, we added the presence of DD as an explanatory factor to adjust for the confounding effect of disc degeneration. Moreover, the presence of buttock and leg pain was added as a dependent variable to the multivariable regression model. LBP is defined as pain in the area bounded by the lowest palpable ribs superiorly and the gluteal folds inferiorly [30]. In this way, participants complaining of buttock pain due to radicular pain might be included in the group of patients with LBP. To adjust for the overlap, the presence of buttock and leg pain was also added as a dependent variable to the multivariable regression model. Finally, adjusting for buttock and leg pain would reinforce the hypothesis that LBP caused by constriction of the dural tube has a different pathology from that of radicular back pain.

After adding these two variables (DD, buttock and leg pain), a narrow CSA of the dural sac was still a significant variable associated with LBP. This result supports the hypothesis that a narrow CSA in the dural sac might be one of the reasons for LBP in LSS patients. Moreover, after adding DD and buttock and leg pain as dependent variables, the AUCs of the ROC curves for the multiple logistic regression analysis increased compared to the AUC before adding dependent variables. Adding the AUC for the ROC curve to the multiple logistic model after adding the presence of DD and buttock and leg pain was 0.66, which was not large. It is assumed that this small value indicates that the CSA of the dural sac might not be strongly correlated with LBP, because LBP can be caused by multiple factors, including osteoporosis, back muscle strain, poor alignment, and psychosocial difficulties. We could explain only a portion of the associated factors for LBP with one factor. However, adding some other factors to the models (MRI findings such as degenerative degeneration, or clinical findings such as buttock and leg pain) yielded a better multivariate model for LBP. Future investigations should include continued follow-up surveys of other factors, such as facet arthropathy or end-plate change, which would enable us to explain more about nonspecific low back pain.

The present study has several limitations. First, although more than 800 participants were included in the present study, the study population may not be representative of the general population because participants were recruited from only one area of Japan. Anthropometric measurements were compared between the participants of the present study and those of the general Japanese population [31]. There was a significant difference in BMI for both men and women in our study and that of the general population (BMI [standard deviation] in men: 24.0 kg/m^2^ [3.5 kg/m^2^] vs. 23.4 kg/m^2^ [3.36 kg/m^2^], respectively, p = 0.00; BMI [standard deviation] in women: 22.57 kg/m^2^ [3.62 kg/m^2^] vs. 22.29 kg/m^2^ [3.69 kg/m^2^], respectively, p = 0.031). Therefore, the participants included in this study might have had a different prevalence of LBP or leg pain. However, we believe that the association between the CSA of the dural tube and LBP, which was shown in this study, could be generalized. Second, this is a cross-sectional study, so any causal relationship between symptomatic LSS and physical performance cannot be clarified. The Wakayama Spine Study is a longitudinal survey, so further progress will help elucidate any causal relationships. Third, the configuration of the dural sac was not taken into account. Generally, stenosis of the lumbar spinal canal is divided into the following categories: central stenosis, lateral recess stenosis, and foraminal stenosis. A comprehensive evaluation of spinal stenosis that includes the presence of lateral recess stenosis or foraminal stenosis, and not just the CSA of the dural tube, would be more appropriate for predicting LBP. Finally, the cut-off values for the CSA of the dural sac also posed a problem. In this study, the first and third quartiles of the mCSA were used as the cut-off values for all levels. However, it is inevitable that two nerve roots depart from the cauda at each vertebral level. Thus, it is reasonable to assume a gradually decreasing cut-off value in the distal direction. It might be more appropriate to use different cut-off values for each intervertebral level.

LBP is caused by multiple factors beyond the scope of MRI findings. However, this study clarified that a narrowed CSA of the dural sac was associated with LBP. Although a narrowed CSA might not be strongly correlated with LBP, these findings contribute to our understanding of LBP. Further investigations along with continued follow-up surveys, including additional radiographic findings and psychological and social factors, including occupation, will continue to elucidate the causes of LBP.
